# An Elite Hybrid Particle Swarm Optimization for Solving Minimal Exposure Path Problem in Mobile Wireless Sensor Networks

**DOI:** 10.3390/s20092586

**Published:** 2020-05-01

**Authors:** Nguyen Thi My Binh, Abdelhamid Mellouk, Huynh Thi Thanh Binh, Le Vu Loi, Dang Lam San, Tran Hai Anh

**Affiliations:** 1 The faculty of Information and Technology, Hanoi University of Industry, Hanoi 100000, Vietnam; binhntm@haui.edu.vn; 2 School of Information Communication Technology, Hanoi University of Science and Technology, Hanoi 100000, Vietnam; binhht@soict.hust.edu.vn (H.T.T.B.); loi9a1999nqb@gmail.com (L.V.L.); san1201.bkhn@gmail.com (D.L.S.); anhth@soict.hust.edu.vn (T.H.A.); 3 Tinc-NET/LiSSi Laboratory, Networks & Telecommunications (N&T) Department, University Paris-Est Creteil (UPEC), F-94400 Vitry, France

**Keywords:** minimal exposure path, wireless sensor networks, mobile wireless sensor networks, hybrid particle swarm optimization

## Abstract

Mobile wireless sensor networks (MWSNs), a sub-class of wireless sensor networks (WSNs), have recently been a growing concern among the academic community. MWSNs can improve network coverage quality which reflects how well a region of interest is monitored or tracked by sensors. To evaluate the coverage quality of WSNs, we frequently use the minimal exposure path (MEP) in the sensing field as an effective measurement. MEP refers to the worst covered path along which an intruder can go through the sensor network with the lowest possibility of being detected. It is greatly valuable for network designers to recognize the vulnerabilities of WSNs and to make necessary improvements. Most prior studies focused on this problem under a static sensor network, which may suffer from several drawbacks; i.e., failure in sensor position causes coverage holes in the network. This paper investigates the problem of finding the minimal exposure paths in MWSNs (hereinafter MMEP). First, we formulate the MMEP problem. Then the MMEP problem is converted into a numerical functional extreme problem with high dimensionality, non-differentiation and non-linearity. To efficiently cope with these characteristics, we propose HPSO-MMEP algorithm, which is an integration of genetic algorithm into particle swarm optimization. Besides, we also create a variety of custom-made topologies of MWSNs for experimental simulations. The experimental results indicate that HPSO-MMEP is suitable for the converted MMEP problem and performs much better than existing algorithms.

## 1. Introduction

Wireless sensor networks (WSNs) are nowadays ubiquitous. They are used in various different domains: military, medical, environment, etc. Research on WSNs has tried to optimize sensor network design [1,2,3]; i.e., coverage quality, network lifetime, sensor placement, communication and computation effort, etc. Coverage is one of the most widely studied problems in WSNs. Coverage of a WSN reflects the quality of service (QoS) provided by that WSN; e.g., a high-quality WSN used in a security system may detect unauthorized penetration with probability higher than 99% [4,5,6]. Among the studies on the coverage problem, barrier coverage has drawn tremendous attention from the academic community due to its huge potential in various security applications [7,8,9,10]. To evaluate coverage quality of WSN, especially in barrier coverage problems, a well-known method is using exposure as a metric [11,12,13]. Great effort in studies related to exposure has been made to investigate the minimal exposure path problem. The goal of the minimal exposure path (MEP) problem is to find a penetration path having minimal exposure value from a source point to a destination point in the sensing field. With the knowledge of MEP, the sensor network designers can appraise weaknesses or worst-case coverage paths of a sensor network, since objects moving across the sensing field along this path have the least capacity to be detected. As a result, information of the MEP can be used in optimizing, managing and maintaining WSNs. Measuring exposure is not only useful in the WSN but also in several other fields, such as evaluating the quality of radio signal propagation or manufacturing path-finding robots.

Recently, mobile wireless sensor networks (MWSNs) have been received increasing interest because of a wide variety of potential applications. A MWSN consists of mobile sensor nodes which are equipped with a locomotive unit and can move around after deployment [14,15,16,17]. Sensors can be attached to larger machines like mobile robots or can be a self-contained miniature system with the ability to move to desired areas. Such MWSNs are extremely valuable in situations where traditional static wireless sensor network (SWSN) deployment mechanisms fail or are not suitable; for instance, a hostile environment where sensors cannot be manually deployed, and must be air-dropped. MWSNs also play an important role in homeland security. Sensors can be mounted on vehicles (subway trains, taxis, police cars, fire trucks, boats, etc.) or carried by people (policemen, fire fighters, etc.). These sensors accompany with their carriers in every motion, and can monitor and dynamically patrol the environment (for chemicals, biological matter, wildfires or radiological agents). For example, MWSN can be used in wildfire monitoring applications. The mobile sensors are able to maintain a safe distance from the fire perimeter, and provide updating information to fire fighters that indicate where the perimeter currently is. In other application scenarios, such as atmosphere and under-water environment monitoring, airborne or under-water sensors may move with the surrounding air or water currents [18,19]. The coverage of a MWSN now depends not only on the initial network configurations, but also on the mobility behavior of the sensors. Thanks to the mobility of sensor nodes, MWSNs can improve coverage quality, prolong the network lifetime, optimize use of resources and be relocated very efficiently [20,21,22].

The MEP problem in SWSNs has been intensively studied by the academic community. However, this problem has not yet been efficiently explored and exploited in MWSNs. Motivated by the advantages of MWSNs and the vital role of the MEP problem, this paper investigates the problem of finding the minimal exposure path in a MWSN (hereinafter MMEP). Specifically, given a set of mobile sensor nodes which are randomly deployed in the region of interest (ROI), the goal of the MMEP problem is to find a crossing path from a source point to a destination point such that an intruder moving along this path has the lowest possibility of being detected, meaning that this path has the minimal exposure value. The MEP problem is an optimization problem. Usually, it is converted into numerical functional extreme problem [23]. Due to high dimensionality and non-linearity of the objective function, and the mobility feature of sensors, the MMEP problem is much more challenging than the traditional MEP problem in SWSNs. Hence, we propose to integrate genetic algorithm into particle swarm optimization to form an efficient algorithm named HPSO-MMEP algorithm.

The main contributions of this paper are as follows:

- Formulating the MMEP problem in MWSNs with several different sensing coverage models.
- Devising an elite hybrid particle swarm optimization algorithm, called HPSO-MMEP, which integrates genetic operators into PSO algorithm to significantly improve the performance of original PSO.
- Designing a new individual representation and proposing a strategy for generating individuals called controlled-point (CP) initialization which ensures the diversity of the generated population.
- Conducting numerous experiments in various scenarios to evaluate the performance of HPSO-MMEP. The experimental results are thoroughly analyzed to give insights into the affects of different factors.

The rest of this paper is organized as follows. Related works regarding the MEP problem are discussed in Section 2. Section 3 provides some preliminaries, the formulation of the MMEP problem and the proposed algorithm HPSO-MMEP. Experimental results are considered in Section 4. Finally, Section 5 presents conclusions and future works of the paper.

## 2. Related Works

This section provides the big picture of literature regarding the MEP problem. Different MEP models dependent on various factors, such as types of sensors, deployment strategies, deployment environments and solution approaches, are briefly reviewed.

Regarding SWSNs, a number of studies have focused on the MEP problem with several different approaches: computational geography, grid-based and heuristic/metaheuristic. Using Voronoi diagram has been the most widely used method for the computational geometry approach. Voronoi diagram-based methods and grid-based methods were the earliest methods to address the MEP problem. In [24], Meguerdichian et al. was the first to devise the concept “exposure”, and contended that finding the MEP in WSNs under arbitrary sensor and intensity models is very meaningful for network designers but is an extremely difficult for optimization tasks. The authors found the closed-form solution in the case of polygonal region with a single sensor under some constraints. In the generic case, where the sensing field consists of multiple sensors, the grid-based method was applied. The MEP problem was then focused on by the academic community [12,24,25,26,27,28]. In [27], Djidjev et al. derived the closed-form solution for the MEP problem with a single-sensor field in both unbounded and polygonal regions. This solution completed the open problems left from [24]; i.e., it worked with both bounded and polygonal regions without any constraints. Based on this result, the authors provided an approximation algorithm for calculating MEP in a multiple-sensor field. The main idea was triangulating the sensing field using a Voronoi diagram, and in the scope of each Voronoi cell, the MEP was calculated by the optimal solution in the single-sensor case. To obtain the solution for the whole sensing field, Dijkstra shortest path algorithm was performed in the final one. While this method successfully dealt with closest-sensor field intensity model, it could not be applied for all-sensor field intensity model. In [12], the authors introduced a very similar concept to MEP which was the maximal breach path—a path running from a single source to a destination point across the sensing field, in which the Euclidean distance from any point on the path to the closest sensor is maximized. The authors proved that the maximal breach path must lie on the edges of Voronoi diagram corresponding to the set of sensors. The maximal breach path was then easily calculated using the Dijkstra shortest path algorithm. Both the minimal exposure path and the maximal breach path provide information about the worst-case coverage of the sensor network; however, the minimal exposure path is more generic since it can be used with a variety of network models. In [29], the authors studied the maximal breach path problem; they then proposed the Voronoi diagram-based algorithm for solving the problem. In [13], the authors extended the previous concept of the worst-case path-based coverage to evaluate the coverage of a given network from a global point of view, taking arbitrary paths into account by considering the arbitrary source and destination pairs. They then presented centralized and distributed algorithms that used knowledge from computational geometry; i.e., Voronoi diagram and Delauney triangulation. The works in [26,30,31,32] addressed different MEP models using the common idea of breaking the region of interest into small grids. In [26], the region of interest was broken into Voronoi cells and the calculation of the MEP was limited to the scope of each cell. The authors then proposed a localized algorithm that can effectively reduce the communication and computation performed by the sensor network. Song et al. [31] and Liu et al. [32] used uniform square grids to partition the ROI. While almost grid-based methods used the Dijkstra shortest path algorithm to search for the MEP, the authors in [31] proposed a metaheuristic algorithm called Physarum optimization algorithm to achieve the minimal exposure road-network among multiple points of interest. This algorithm then can be applied to solve the Steiner tree problem. In [32], the authors used percolation theory to derive the critical conditions for the existence exposure path, where an exposure path is a penetration path through the sensor network on which every point is not covered by any sensor.

The advantage of the Voronoi diagram-based method is that it extracts the geometric characteristics of Voronoi diagram to reduce the significant computational effort. However, this method only works under a particular type of network model. Unless the network is homogeneous and the sensors are isotropic, the Voronoi diagram-based method cannot obtain the solution because if not for that case, the intrinsic geometric characteristics of Voronoi diagram will no longer reflect information about the MEP. For the grid-based method, its main idea can be summarized as follows: (1) transform the continuous search domain of the MEP problem into a discrete one by discretizing the region of interest into small grids; (2) construct a graph based on these grids and perform a graph search algorithm to calculate MEP. The grid-based method can deal with almost all types of network model, and different constraints of the particular problem. However, the results of this method are limited; i.e., the path that is found always follows fixed directions, which does not follow realistic scenarios. In addition, there must be a trade-off between the solution accuracy and the computation effort; i.e., to get higher accuracy, the grid size must be smaller, which increases the computational time.

Since the Voronoi diagram-based and the grid-based methods posed several disadvantages, as mentioned above, recently, heuristic/metaheuristic methods which were inspired by the process in nature, such as particle swarm optimization (PSO) and the genetic algorithm, were applied to solving the MEP problem [29,33,34,35,36,37]. These studies converted the MEP problem into the numerical functional extreme (NFE) problem [23] by breaking the penetration path into small enough path intervals and then performed a metaheuristic algorithm to search for the optimal result. Although the general idea was shared among these studies, each work proposed different techniques that are appropriate to the particularities of the models considered. Miao et al. [33] modified the PSO algorithm by adding a Gauss mutation operator and a projection operator to avoid the saw-tooth shape of the obtained path. To eliminate this saw-tooth effect of the metaheuristic algorithm and improve the efficiency, the authors in [34] proposed a genetic algorithm with a special crossover operator, a local search scheme and an upside-down operator. Feng et al. [37] considered the MEP problem with path constraints. A genetic algorithm was then proposed with a local search operator that could effectively remove the saw-tooth shape in the path solution and increase the convergence speed. Binh et al. [35] studied the MEP problem under a practical probabilistic coverage model, which took environmental factors into consideration and regarded them as noise. The problem was then addressed using a genetic algorithm with two efficient crossover operators and one mutation operator. The authors also investigated the MEP problem under a directional heterogeneous sensor network in [36]. This work introduced a novel PSO algorithm based on gravity force theory. All of those studies on the MEP problem were done to achieve a solution as good as possible in the least computational time. Compared to previous Voronoi diagram-based and grid-based approaches, this approach obtained better results in less computational time.

We have delved into the related works of the MEP problem in WSNs. It is certain that almost the authors did not regard moving ability of sensor nodes. Previously, there was only one research paper related to MMEP, written by Zhang et al. [38]. Nevertheless, their study solved the MEP problem under a hybrid sensor network with both static and mobile sensor, in which the minimal exposure path under static sensors that could avoid mobile sensors was introduced. The approach of this work was a Voronoi diagram method which could not be generalized to deal with other network models, such as an entirely mobile sensor network or a heterogeneous sensor network. Our previous publication in 2017 [39] also studied the MEP problem in MWSNs. However, the research result was not optimal, which served as the motivation for us to do further research and come up with a more efficient solution for the MMEP problem. Therefore, in this paper, we introduce a heuristic strategy called control-point initialization and propose the HPSO-MMEP algorithm to effective solve the MMEP problem.

We summarize all the related works presented in this section in Table 1.

## 3. Proposed Algorithm: HPSO-MMEP

### 3.1. Preliminaries

#### 3.1.1. Sensing Model

A sensing model describes the ability of a sensor node to sense its surrounding environment. In reality, almost all types of sensors share a feature in common: the sensing quality, or sensing intensity, of a sensor decreases as the distance away from the sensor increases. In this paper, we adopt a widely used sensing model called *attenuated disk model* and another variant called *truncated attenuated disk model*. Let f(l,p) be the sensing intensity function of the sensor located at the position *l* on the target point *p*; the expression of f(l,p) for the attenuated disk model is:

(1) $$f\left(l,p\right)=\frac{C}{d^{\lambda }\left(l,p\right)}$$

where d(l,p) is the Euclidean distance between the sensor and the the target point *p*; *C* is a constant depending on the essence of sensor; λ is the attenuation exponent, which depends both on the sensor and the environment. For the truncated attenuated disk model, the sensing intensity function is:

(2) $$f\left(l,p\right)=\left\{\begin{array}{cc} 1 & ,\mathrm{if}\;d\left(l,p\right)\leq R_{1}\\ e^{-\alpha {\left[d\left(l,p\right)-R_{1}\right]}^{\beta }} & ,\mathrm{if}\;R_{1}<d\left(l,p\right)\leq R_{2}\\ 0 & ,\mathrm{otherwise} \end{array}\right.$$

where α and β are constants, $R_{1}$ is a certain range, $R_{2}$ is a sensing range and $R_{2}-R_{1}$ is an uncertain range.

**Figure 1:**
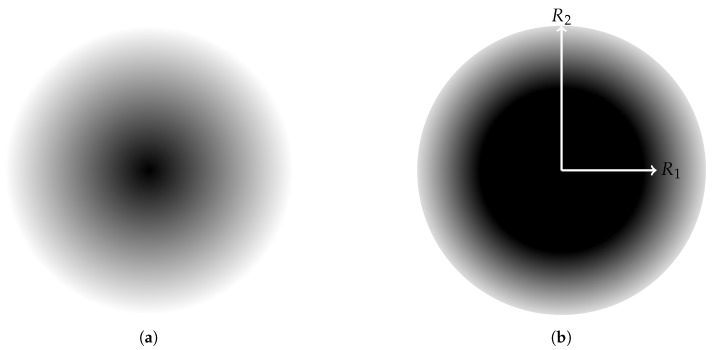
Illustration of (**a**) an attenuated disk model; (**b**) a truncated attenuated disk model.

Figure 1 illustrates such two attenuated sensing models.

#### 3.1.2. Sensor Field Intensity Model

The sensor field intensity model specifies the collaboration of sensors in the sensing field. In this paper, we use the all-sensor field intensity model, which is described as follows:

(3) $$I\left(p\right)=\sum_{i=1}^{N}f\left(l_{i},p\right)$$

where *N* is the number of sensor nodes in the sensor network; i.e., the sensor network consists of *N* sensors $\left\{s_{1},s_{2},\ldots ,s_{N}\right\}$. $l_{i}$ is the location of sensor $s_{i}$; *p* is the target point.

#### 3.1.3. Exposure

The concept of exposure was introduced by Meguerdichian et al. [24] as a metric to measure how well an object moving on an arbitrary path can be observed by the sensor network. According to that, the exposure of an object *O* in the sensing field during time interval $\left[t_{1},t_{2}\right]$ along the path p(t) is defined as:

(4) $$E\left(p\left(t\right),t_{1},t_{2}\right)=\int_{t_{1}}^{t_{2}}I\left(p\left(t\right)\right)\left|\frac{dp(t)}{dt}\right|dt$$

In SWSN, the sensing intensity at a specific position does not depend on time, which reduces I(p(t)) to I(p) only. The expression $\left|\frac{dp(t)}{dt}\right|dt$ can be replaced by dp, which represents a path element. To avoid ambiguity, we denote the object trajectory as P instead of p(t). Then, Equation (4) can be rewritten as follows:

(5) $$E\left(P\right)=\int_{P}I\left(p\right)dp$$

Equation (5) means that exposure of an object *O* along path P does not depend on time but only the geometric characteristic of P. Equation (5) can be interpreted as the accumulation of sensing intensity over all points on the path P.

In MWSN, Equation (5) cannot be applied to calculate the exposure value due to several problems: (1) The sensing intensity at a specific position changes over time. (2) One would expect the exposure value of an object *O* through the sensor network to get higher if the time for which *O* stays in the sensing field is longer. Suppose *O* freezes for a time interval ϕ; then Equation (5) causes exposure of *O* in ϕ equal to zero, which is unreasonable in reality. Exposure value should be formulated as the accumulation of sensing intensity over every state of the object. While the position, or the coordinates, determines the state of an object in SWSN, time is the representation for the state of an object in MWSN. Therefore, in this paper, we use the exposure formula defined by Binh et al. [39], which is:

(6) $$E\left(P,T\right)=\int_{0}^{T}I\left(p\left(t\right)\right)dt$$

Here, we assume that the object always enters the sensing field at t=0 and exits at t=T.

### 3.2. Problem Formulation

Finding an analytical solution for the MEP problem is a functional extreme problem in mathematics, which is commonly solved by using Euler–Lagrange differential equation. However, this method works only for several simple cases, where the solution of Euler–Lagrange differential equation can be obtained easily. The authors in [27] derived the exact solution for the MEP in the presence of a single sensor, but none of existing works can provide a closed-form expression for the MEP in multiple sensor scenario. Therefore, this paper investigates to find an approximate solution for the MMEP problem by converting the continuous domain of the problem to a discrete one. Specifically, time interval [0,T] for which an object *O* stays inside the sensing field is partitioned into a set of finite sub-intervals with the same length Δt. Δt should be small enough such that sensing intensity does not vary too much between two consecutive moments. Then, the right side of (6) can be approximated as follows:

(7) $$\int_{0}^{T}I\left(p\left(t\right)\right)dt\approx \sum_{i=1}^{m}I\left(p\left(i\Delta t\right)\right)\Delta t,\;\mathrm{with}\;m=\left\lfloor \frac{T}{\Delta t}\right\rfloor$$

The MMEP problem can be briefly stated as follows: given a mobile sensor network with *N* sensor nodes $\left\{s_{1},s_{2},\ldots ,s_{N}\right\}$ randomly distributed in the ROI Ω. The sensor $s_{i}$ has the initial location $l_{i}$, and the location at time *t* is denoted as $l_{i}\left(t\right)$. Each sensor moves in a preset trajectory at constant speed. Any object penetrating the monitoring region always starts from the source point *S* and goes to the destination point *D*. The time interval for which the object stays inside the sensing field is [0,T]. Speed of the object is limited at a maximum value, and we assume that the object always moves at its maximum speed, since exposure will get higher if the object stays longer in the sensing field. The goal of the MMEP problem is to find out a penetration path P from the beginning point *S* to the ending point *D* such that the exposure value of the path P is minimal. More precisely, the MMEP problem is formulated as follows:

**Input:**

- W,H: the length and the width of the ROI Ω.
- $\left(0,y_{S}\right)$: coordinates of the source point *S*.
- $\left(W,y_{D}\right)$: coordinates of the destination point *D*.
- *N*: number of mobile sensor nodes.
- $\left\{s_{1},s_{2},\ldots ,s_{N}\right\}$: set of sensor nodes deployed in Ω.
- $l_{i}$: the initial location of sensor $s_{i}$, $i=\bar{1,n}$.
- $R_{i}$: trajectory of sensor $s_{i}$.
- $v_{s}$: speed of sensor nodes, all sensor nodes have the same speed.
- $v_{I}$: maximum speed of the intruder.

**Output:**

A path P connects the source point *S* to the destination point *D*.

**Objective:**

Exposure of P is minimum, which means:

(8) $$\sum_{i=1}^{m}I\left(p\left(i\Delta t\right)\right)\Delta t\to \min,\;m=\left\lfloor \frac{T}{\Delta t}\right\rfloor$$

**Constraint:**

- An intruder always moves towards the right side of Ω; i.e., the distance from the intruder to the right border of Ω gets smaller over time.
- The intruder always moves at its maximum speed $v_{I}$

### 3.3. Proposed Algorithm

The objective function (OF) of the formulated MMEP problem strongly depends on sensing intensity function I(.), which is specified by sensing model and sensor field intensity model. Commonly, OF is very complicated; i.e., it is non-linear, high-dimensional and non-differential. A deterministic optimization approach like steepest descent or the interpolation method is infeasible since such methods require one to efficiently compute the derivative value of OF. A potential approach to deal with such complicated OF is to use metaheuristic algorithms. Metaheuristics are general algorithmic ideas that can be applied to solve a board range of optimization problems without depending on the particularity of the problem. There are many different existing metaheuristic algorithms and they can be used in combination to achieve better performance. In this paper, we integrate the genetic algorithm (GA) into particle swarm optimization (PSO) to form the HPSO-MMEP algorithm. While PSO is benefited from the collaboration of biological populations and can rapidly converge to optimal solution, GA keeps the population from getting trapped in local optima by using genetic operators. The following subsections, HPSO-MMEP will be discussed in detail.

#### 3.3.1. Individual Representation

**Figure 2:**
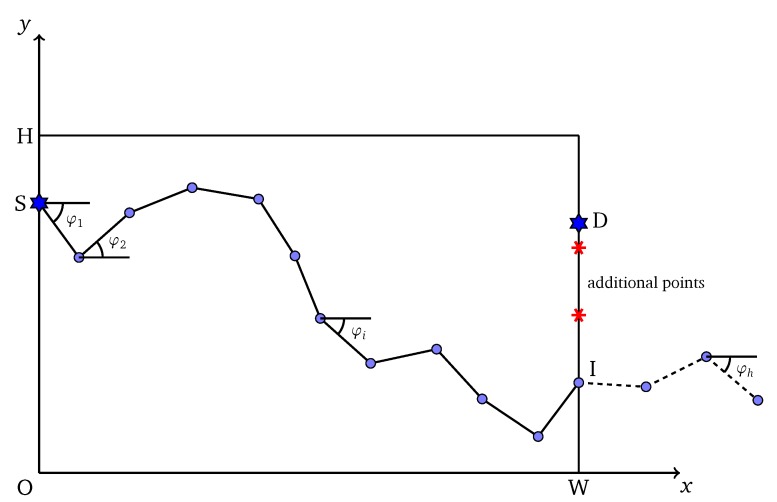
Individual representation for HPSO-MMEP.

An individual corresponds to a solution of the problem. In this problem, an individual is illustrated as a list of angles: $L=\{\varphi_{1},\varphi_{2},\ldots ,\varphi_{h}\}$ with fixed size *h*, where an angle of a line is the angle between that line and the horizontal axis Ox. A penetration path of solution is formed by the combination of multiple line segments with the predefined length Δs and angles taken from *L*. The predefined Δs is also called the interval length of the path and the value Δs should be small enough to assure the accuracy of HPSO-MMEP. Figure 2 illustrates the individual representation in HPSO-MMEP. Note that the operator of PSO requires the individual to be at the same size or the same number of angles to be performed. Therefore, the size *h* of *L* is fixed at a value that guarantees the formed path P shall at least reach the right border of the monitoring region Ω. The intersection point between P and the right border might not be the destination point *D* and P might go over the right border (dash line in Figure 2). However, the path is restricted to the scope 0≤y≤H.

To calculate exposure of a penetration path represented by *L*, we ignore proportion of *L* which corresponds to the dash line in Figure 2—i.e., the proportion of the penetration path that goes over the ROI Ω. After that, points with equal distance Δs are added to the line segment connecting *I* and *D*, where *I* is the intersection point between the penetration path and the right border of Ω. The distance between *D* and its consecutive point is equal to or less than Δs. Finally, the path connecting S,I,D is used to calculate the exposure of an individual represented by *L*. An intuitive description about this is shown in Figure 2.

#### 3.3.2. Control-Point Initialization

The random initialization method often fail to generate diverse individuals; thus, a heuristic is added to surpass this drawback. We design a new initialization strategy called *control-point* initialization to improve the diversity of the population and the search space of the algorithm. Details of the control-point initialization method are as follows:

1. Partition the ROI Ω along Ox axis into multiple sub-regions with random size. On each boundary between these sub-regions, randomly choose a point, called *control point*. Note that the number of control point is random.
2. Sequentially generate angles according to a probability distribution K. The probability distribution is expected to drive the next angle toward to current control point (the left-most control point).
3. If the current sub-path, which consists of line segments corresponding to generated angles, reaches the right border, sample the remaining angles, i.e., until the number of angles equal to *h*, according to uniform distribution $U\left(\frac{-\pi }{2},\frac{\pi }{2}\right)$ (totally random). Note that the destination point *D* is considered as the last control point.

**Figure 3:**
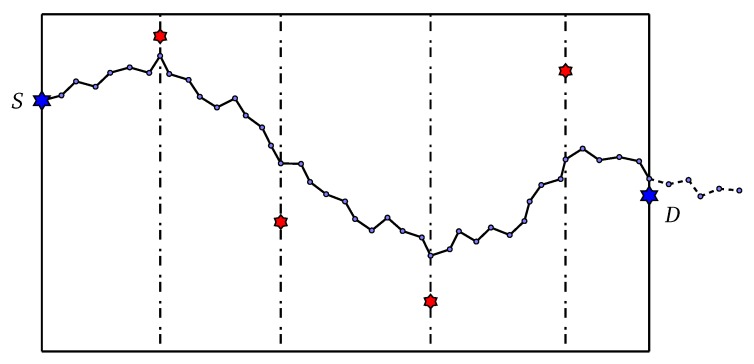
Red stars are the control points that drive the path.

Figure 3 depicts our proposed control-point initialization method.

There might be many different probability distributions that can be used for the generation of angles. We briefly describe the probability distribution used in this paper as follows.

**Figure 4:**
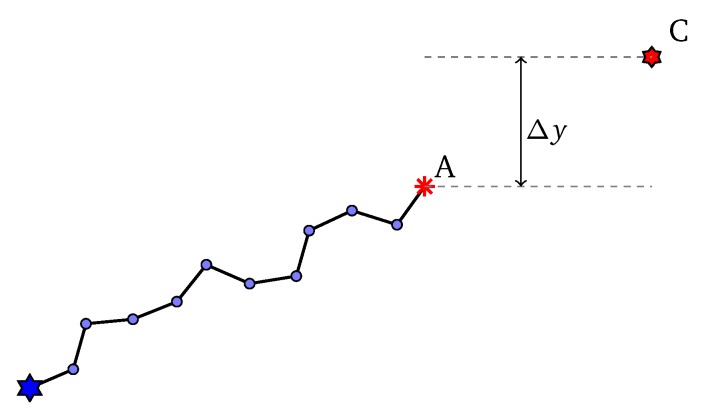
Illustration of sample an angle.

Supposed that at some steps of the initialization process, we are sampling the next angle φ, the current sub-path reaches point *A* and the current control point is *C* (Figure 4). Let ΔPr=Pr(φ≥0)−Pr(φ<0), $\Delta y=y_{C}-y_{A}$. In a uniform distribution, we always have Pr(φ≥0)=Pr(φ<0)=0.5. However, to make the control points able to drive the path, we establish a dependency of ΔPr on Δy. We should all agree to the following intuition:

(9) $$\Delta \mathrm{Pr}=\left\{\begin{array}{cc} 0, & \mathrm{if}\;\Delta y=0\\ 1, & \mathrm{if}\;\Delta y=H \end{array}\right.$$

**Figure 5:**
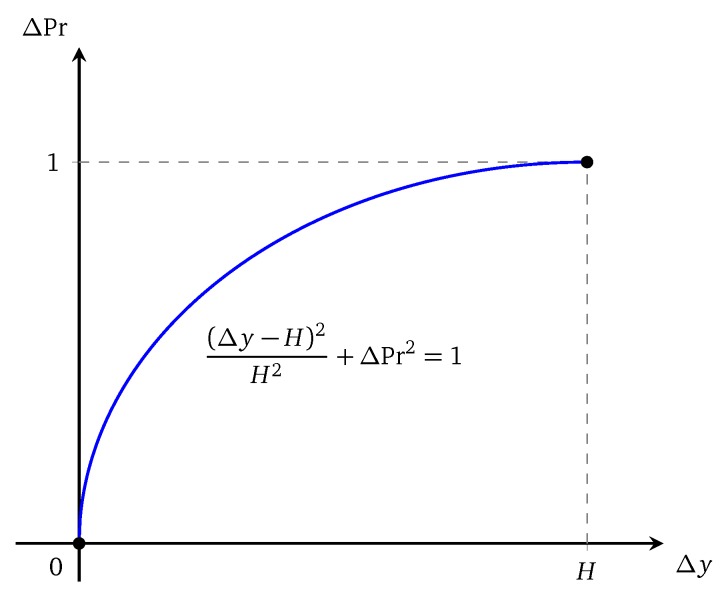
Dependency of ΔPr on Δy.

There is no need to consider negative value of ΔPr here because it is completely symmetric to the positive one. We observe that linear dependency between ΔPr and Δy makes the control points less important because the probability that the path orients toward the control point is not high enough. Therefore, we use a non-linear mapping as in Figure 5.

Let ${\mathrm{Pr}}_{u}$ and ${\mathrm{Pr}}_{d}$ stand for Pr(φ≥0) and Pr(φ<0). Then we have:

(10) $$\left\{\begin{array}{c} {\mathrm{Pr}}_{u}+{\mathrm{Pr}}_{d}=1\\ {\mathrm{Pr}}_{u}-{\mathrm{Pr}}_{d}=\Delta \mathrm{Pr}=\sqrt{1-\frac{{\left(\Delta y-H\right)}^{2}}{H^{2}}} \end{array}\right.$$

From this set of equations, ${\mathrm{Pr}}_{u}$ and ${\mathrm{Pr}}_{d}$ are calculated and the probability distribution is determined. In case Δy<0, simply an inverse sign of ΔPr can give the result.

From this initialization method, it can be expected that the generated individuals will be more diverse, as the paths are able to reach the far top or bottom of the region. Therefore, the population can exploit a larger region and the search space will be enlarged as well.

#### 3.3.3. Integration of GA into PSO Algorithm to Form HPSO-MMEP

The original PSO algorithm can be mapped to the process of a bird swarm seeking food. Individuals in the swarm collaborate and use their historical experiences to get better positions. After a while, the swarm might enter a local optima trap and almost be unable to escape, since the convergence degree at the time is quite high, or, stated differently, the searching space is remarkably shrinking. This is the right time for genetic operators to perform. The crossover and mutation operators of the genetic algorithm can deal with a multiple local optima objective function since they can efficiently expand the searching space and maintain the diversity of the population. HPSO-MMEP is formed by periodically performing genetic operations on the population during PSO search. The update operator of HPSO-MMEP is basically the same as in the original PSO. We recall the update operator in Algorithm 1.

**Algorithm 1:** PSO update operator
 **Input**:
 The individual at step *t*: $L^{t}=\left\{\varphi_{1}^{t},\varphi_{2}^{t},\ldots ,\varphi_{h}^{t}\right\}$
 **Output**:
 The updated individual at step t+1: $L^{t+1}=\left\{\varphi_{1}^{t+1},\varphi_{2}^{t+1},\ldots ,\varphi_{h}^{t+1}\right\}$
1 Veclocity update
2 $v^{t+1}=\omega *v^{t}+r_{1}*C_{1}*\left({\mathrm{pBest}}_{L}^{t}-L^{t}\right)+r_{2}*C_{2}*\left({\mathrm{gBest}}^{t}-L^{t}\right)$
3 Position update
4 $L^{t+1}=L^{t}+v^{t+1}$
5 pBest update
 /∗ $pBest_{L}$ is the best position found by individual *L*∗/ /∗ or the best individual among {$L^{1},L^{2},\ldots ,L^{t}\}$∗/6 **if**
*L is better than $pBest_{L}$*
**then**
7 $\;|\;pBest_{L}\leftarrow L$
8 **end**
9 gBest update
 /∗ gBest is the best position found by the population∗/ /∗ or the best individual among all pBest∗/10 **if**
*L is better than gBest*
**then**
11  | gBest←L
12 **end**


$r_{1},r_{2}$ are random numbers in range (0,1); ω, $C_{1}$, $C_{2}$ are acceleration coefficients of PSO that depend on particular problem. These coefficients are usually obtained from experiments.

For steps at which genetic operators are used, such operators are performed after the PSO update operation. The period of using genetic operators should be configured at an appropriate value such that the population can benefit from the PSO search process. The period is the time for the population to reach a certain convergent level by performing a PSO update operation. If we use genetic operators at every step, the effects of genetic operators may dominate the effects of PSO update operation because genetic operators make the whole population change remarkably.

#### 3.3.4. Crossover Operator

**Figure 6:**
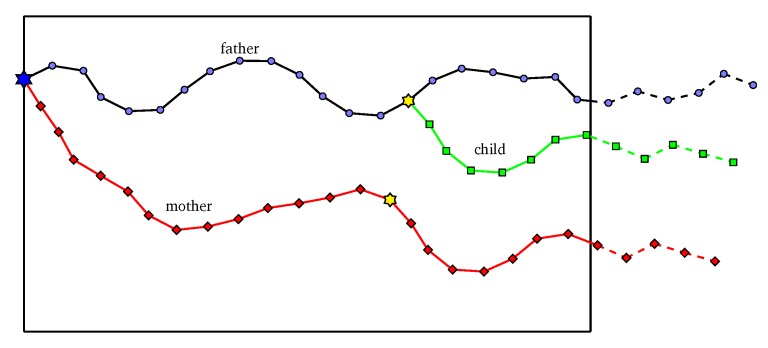
Illustration of the crossover operator of HPSO-MMEP.

The crossover operator is a binary operator. It takes two operands corresponding to two individuals in the population. Figure 6 demonstrates the crossover operator of HPSO-MMEP algorithm. It can be described in the two steps as follows:

1. Choosing a position to perform the operator by sampling a random integer *k* in {1,2,…,h}. Recall that *h* is the number of genes of each individual in the population.
2. Concatenating the beginning part of a father individual from 1 to k−1 and the ending part of mother individual from *k* to *h* to form the child individual.

If the child is better than both of its father and mother, the father will be replaced by the child. If the child is better than one of its parents, that parent will be replaced by the child. Otherwise, nothing changes. Note that when an individual changes, we need to update its pBest and gBest of the population.

#### 3.3.5. Mutation Operator

**Figure 7:**
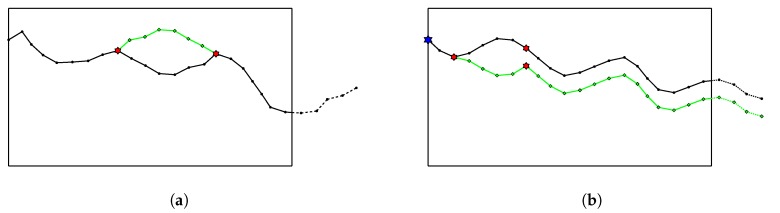
Mutation operators: (**a**) Inverse mutation. (**b**) Symmetric mutation.

We use two mutation operators, which are *inverse mutation* and *symmetric mutation*. The *inverse mutation* is obtained by randomly choosing a continuous set of genes and inverse the order of elements in that set. The *symmetric mutation* shares the same part of choosing genes in common, but instead of inverting the order of those genes, we invert the sign of each gene in the set. The results are shown in Figure 7.

While the *inverse mutation* results in a relatively similar individual to the original one, *symmetric mutation* creates a significantly different individual. *Inverse mutation* can also be seen as local searching that tries to search for a better solution in neighborhood region. *Symmetric mutation* greatly expands and explores the searching space, and thus, it has potential to find the global optimum.

#### 3.3.6. Complexity Analysis

In this section, we analyze time complexity of our HPSO-MMEP algorithm by evaluating the time complexity of calculating the objective function. The value of the objective function of an individual is also called the *fitness* of that individual. Each individual is composed of *h* genes, corresponding to *h* path intervals with equal length Δs. The fitness of an individual *L* is calculated by summing up the fitness of each of its genes. For calculating the fitness of a gene, or a path interval Δs, the algorithm must traverse through all sensors in the sensing field; this requires time O(N), where *N* is the number of sensors. Therefore, time complexity for calculating the fitness of an individual is O(h∗N).

As aforementioned in Section 3.3.1, the fitness of an individual is calculated by an alternative individual, which corresponds to the path connecting S,I,D in Figure 2. Assume Δs is constant, we prove that length *h* of the alternative individual is O(W+H).

**Proof.**  Consider a path with two components: the sub-path from the source point *S* to the right border of the ROI Ω and the vertical sub-path towards the destination point *D* (the path connecting S,I and the path connecting I,D in Figure 2 respectively). For the latter sub-path, denote the number of genes as *t*. Then, *t* is equivalent to the number of additional points described in Section 3.3.2, and it is obviously true that $t=O\left(\frac{H}{\Delta s}\right)=O\left(H\right)\left(*\right)$.

For the former sub-path, we estimate the probability distribution of its length as follows.

Denote L as length of this sub-path and *g* as the number of its genes; then we have:

(11) $$\begin{array}{cc} L & =\Delta s*g\\ W & =\Delta s\left[\cos\left(\varphi_{1}\right)+\cos\left(\varphi_{2}\right)+\ldots +\cos\left(\varphi_{g}\right)\right] \end{array}$$

The probability distribution of a gene φ has already been presented in Section 3.3.2. According to that, φ is equal to the multiplication of two independent random variables *A* and α, where *A* determines the sign of φ and α determines the magnitude of φ. Specifically, $\alpha ∼U(0,\frac{\pi }{2})$ and *A* follows a discrete probability distribution: Pr(A=1)=Pr(φ≥0), Pr(A=−1)=Pr(φ<0). Based on this, we can easily determine the probability distribution of |φ| as follows:

(12) $$\left|\varphi |=|A*\alpha |=|A|*|\alpha |=A∼U(0,\frac{\pi }{2}\right)$$

Let Z=cos(|φ|); we can compute the expectation μ(Z) and standard deviation σ(Z) of *Z*. We only present here the final results:

(13) $$\mu \left(Z\right)=\frac{2}{\pi },\;\sigma \left(Z\right)=\frac{1}{2}-\frac{4}{\pi^{2}}$$

We perform some transformations on the second line of Equation (11):

(14) $$\begin{array}{cc} W & =\Delta s\left[\cos\left(\varphi_{1}\right)+\cos\left(\varphi_{2}\right)+\ldots +\cos\left(\varphi_{g}\right)\right]\\ \; & =\Delta s\left[\cos|\varphi_{1}\left|+\cos\right|\varphi_{2}\left|+\ldots +\cos\right|\varphi_{g}|\right]\\ \; & =\Delta s(Z_{1}+Z_{2}+\ldots +Z_{g})\\ \; & =\Delta s*g*\bar{Z}\\ \; & =L*\bar{Z} \end{array}$$

where $Z_{1},Z_{2},\ldots ,Z_{g}$ are *g* independent random variables with the same probability distribution as *Z*, $\bar{Z}=\frac{Z_{1}+Z_{2}+\ldots +Z_{g}}{g}$. Assume that W≫Δs; then g≫1. According to the central limit theorem, probability distribution of $\bar{Z}$ can be approximated by the normal distribution $N\left(\mu \left(Z\right),\frac{\sigma^{2}\left(Z\right)}{g}\right)$. For a normal distribution $N(\mu ,\sigma^{2})$, 99.7% of the observations are within [μ−3σ,μ+3σ]. Hence, the probability that $\bar{Z}$ is within $\left[\mu \left(Z\right)-\frac{3\sigma (Z)}{\sqrt{g}},\mu \left(Z\right)+\frac{3\sigma (Z)}{\sqrt{g}}\right]$ is 99.7%. Since g≫1 and σ is small $\left(\frac{1}{2}-\frac{4}{\pi^{2}}\right)$, in practice, we can assume that value of $\bar{Z}$ is bounded within a specific interval, and hence, from Equation (14), we have L=O(W), which deduces to $g=\frac{L}{\Delta s}=O\left(W\right)\left(**\right)$.

From (∗) and (∗∗), we have h=t+g=O(H)+O(W)=O(W+H), which is what must be proven. □

To conclude, time complexity of our HPSO-MMEP algorithm is $O\left((W+H)*N\right)$.

## 4. Experimental Results

This section investigates effects of important factors on the performance of the proposed HPSO-MMEP algorithm. To prove the efficiency of HPSO-MMEP, we compare the algorithm with two existing algorithms, which are GAMEP [39] and HPSO [33], in various experimental scenarios. In the last case, effects of several factors relating to the MMEP problem such as speed of intruder $v_{I}$, number of sensors *N* and network model (mobile/static) are thoroughly analyzed.

### 4.1. Experimental Settings

#### 4.1.1. Data Settings

We establish eight different experimental scenarios. For each scenario, the number of sensors *N* varies in {25,50,75,100}, which in turn contains 10 topologies. Since HPSO-MMEP is approximate algorithm, we run on each topology 20 times to get average value and measure the standard deviation. A scenario is determined by a sensing model, a sensor trajectory and a sensor distribution, which are as follows:

**Sensing model**

- Attenuated disk model
- Truncated attenuated disk model

**Sensor trajectory**

- Random point trajectory.
- Rectangle trajectory.

**Figure 8:**
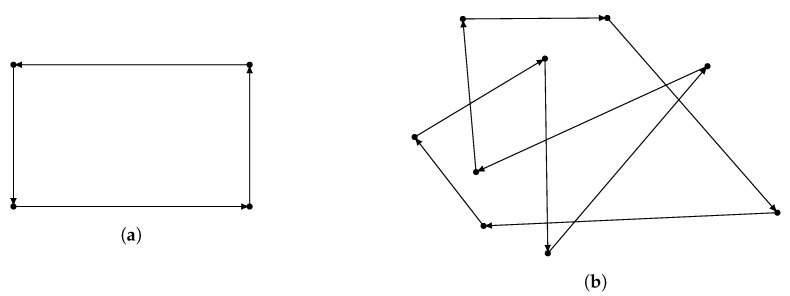
Sensor trajectory: (**a**) Rectangle trajectory. (**b**) Random point trajectory.

Sensor trajectory is characterized by a set of fixed positions, and sensor will move from one position to the next in that set. The movement repeats when the sensor reaches the last position in the position set [17]. Rectangle trajectory corresponds to a position set with four points that are four corners of a rectangle. Random point trajectory corresponds to a random-size set of random positions. Figure 8 illustrates these two sensor trajectory.

**Sensor distribution**

- Uniform distribution.
- Gauss distribution.

Differently from SWSNs, sensors in MWSNs change their positions over time. Hence, sensor distribution in MWSNs refers to the distribution of every position in the position set of every mobile sensor.

An experimental instance corresponds to an experimental scenario with a specific number of sensors and is named in the format “*Dis*_*Model*_*Traject*_*Num*” in which *Dis* presents the sensor distribution: “*u*” for uniform and “*g*” for Gauss; *Model* is the sensing model: “*a*” for attenuated and “*t*” for truncated attenuated; *Traject* is the trajectory of sensor: “*ran*” for random point and “*rec*” for rectangle; “*Num*” is the number of sensor. Although 10 topologies are generated per a specific number of sensors per a scenario, we select only one topology for experiments presented in later sections and do not regard the topology in an experimental instance.

#### 4.1.2. Parameters and System Settings

All subsequent sections share the following parameters: W=100 m, H=40 m, the source point S=(0,30), the destination point D=(100,10), the speed of sensors $v_{S}=1$ and the speed of intruder $v_{I}=2$. The sensor network is homogeneous and entirely mobile. A position of a sensor is pair of independent random variables (X,Y). For uniform distribution, X∼U(0,W) and Y∼U(0,H), where U(a,b) denotes uniform distribution on interval [a,b]. For Gauss distribution, X∼U(0,W) and $Y∼N(\mu ,\sigma^{2})$ where $N(\mu ,\sigma^{2})$ is Gauss distribution with expectation μ and standard deviation σ. The following are settings of the Gauss distribution used in all our experiments:

- For rectangle trajectory, $\mu =y_{S}=30$ and $\sigma^{2}=200$ for all positions in the position set of all sensors.
- For random point trajectory, $\mu =y_{S}=30$ and $\sigma^{2}=64$ for initial position of all sensors, $\mu =y_{S}=30$ and $\sigma^{2}=200$ for all other positions in the position set of all sensors.

Configurations of attenuated and truncated attenuated sensing models are given in Table 2 and are applied to all sensors.

Parameters settings for HPSO-MMEP algorithm are shown in Table 3.

All our experiments were run on a machine with Intel^®^Core™i7-4790 CPU at 3.60 GHz 16 GB RAM using the Java language.

### 4.2. Computation Results

#### 4.2.1. Effects of Important Factors on the Performance of HPSO-MMEP

*(a) Performance of HPSO-MMEP using different Δs* ($\Delta s=v_{I}\Delta t$) 

**Figure 9:**
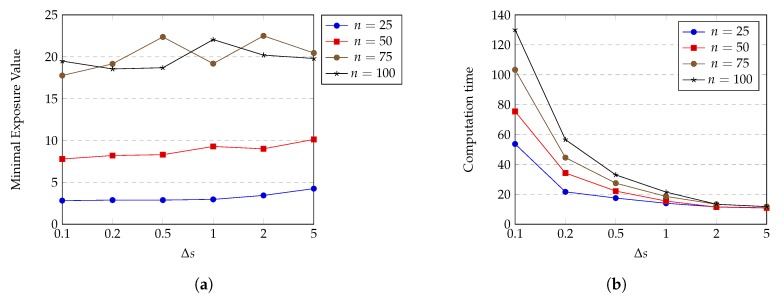
Effect of Δs on (**a**) minimal exposure value; (**b**) computation time.

Δs does not appear in exposure calculations; however, it still reflects the accuracy of the exposure approximation; i.e., transform the integral form of exposure $E\left(P,T\right)=\int_{0}^{T}I\left(p\left(t\right)\right)dt$ to sigma sum form $E\left(P\right)=\sum_{i=1}^{m}I\left(i\Delta t\right)\Delta t$. We have $\Delta t=\frac{\Delta s}{v_{I}}$, and Δt should be at an appropriate value such that sensing intensity does not vary too much on that time interval. The expectation of having similar sensing intensity in Δt can be achieved by choosing small enough value of Δs because points with small distances from each other tend to have similar sensing intensity values. On the other hand, decreasing of Δs causes the exposure calculation to be more complex, thereby increasing the computation time of HPSO-MMEP. Figure 9 shows the effects of Δs on the minimal exposure value and the computation time achieved by HPSO-MMEP algorithm. For this experiment, we examine on the experimental instance *u*_*a*_*rec*.

From Figure 9a, we can see that for a small number of sensors (N=25 and N=50), the minimal exposure value continuously slightly rises when the value of Δs increases from 0.1 to 5. This is caused by two reasons: (1) the large value of Δs results in the lower dimension of the searching space, which limits the searching capability of HPSO-MMEP, and thus, gives a worse solution; (2) the large value of Δs degrades the accuracy of exposure calculation, which increases the error between the approximate exposure value and the true exposure value of a penetration path. However, there is no significant difference in the minimal exposure value at Δs=0.1 (the smallest) and Δs = 5 (the largest). This can be explained that due to uniform distribution and the sparsity of sensors, there exist wide areas in the ROI Ω in which every point has relatively similar and low sensing intensity. Therefore, the large value of Δs still guarantees that sensing intensity does not vary too much on that path interval. In contrast, for a large number of sensors (N=75 and N=100), the minimal exposure value changes significantly and unpredictably when Δs increases. In all cases, we obtain quite stable solution at Δs = 0.1 and Δs = 0.2. Figure 9b indicates that the computation time of HPSO-MMEP is inversely proportional to Δs. When Δs=0.1, computation time jumps up an extremely big distance compared to other values of Δs. Note that too small a value of Δs makes the penetration path not realistic since it is not smooth and has a saw-tooth shape. Based on what we have analyzed so far, we choose the optimal value of Δs to be 0.2 and use this value for experiments in all subsequent sections.

*(b) Performance of HPSO-MMEP in the presence of genetic operators*

**Figure 10:**
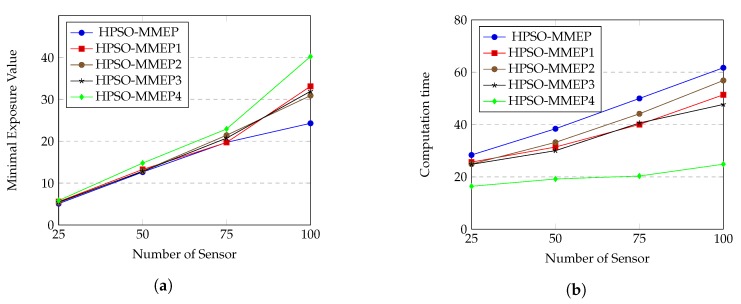
(**a**) Minimal exposure value; (**b**) computation time.

We evaluate the effects of different genetic operators on HPSO-MMEP algorithm by creating different versions of HPSO-MMEP, as shown in Table 4, and perform each version on the experimental scenario *u*_*a*_*rec*. The results are shown in Figure 10.

From Figure 10a, we can see that three versions, HPSO-MMEP1, HPSO-MMEP2 and HPSO-MMEP3, give quite similar minimal exposure values when the number of sensors varies. This means that three genetic operators, which are crossover, inverse mutation and symmetric mutation, tend to give the same effects on the performance of HPSO-MMEP. The computational times of these three versions (Figure 10b) are also not too different. The difference becomes clearer when all the three operators are in used or all of them are unused. For the minimal exposure value, HPSO-MMEP performs best with big number of sensors. This can be explained: when the number of sensors is large, there is little chance of finding a good solution. Hence, expanding the search space by using the genetic operators results in much better solution. However, when number of sensors is small, just using original PSO is enough to get the solution.

*(c) Performance of HPSO-MMEP using control-point initialization method*

**Figure 11:**
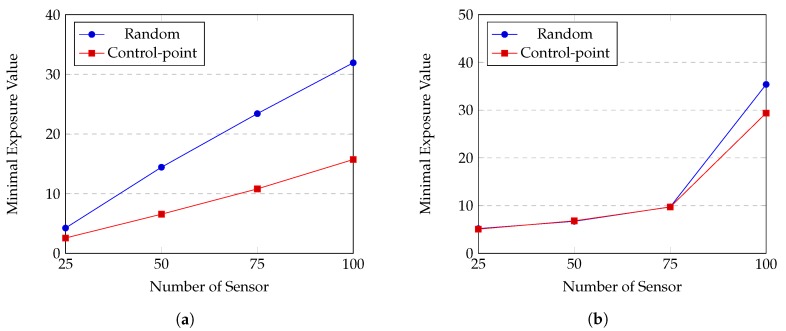
Random vs. control-point initialization: (**a**) Gauss distribution. (**b**) Uniform distribution.

We evaluate effect of the control-point initialization by comparing it with random initialization. The experiments are conducted on Gauss and uniform distributions of sensors, corresponding to Figure 11a,b respectively. The trajectory of the sensor is random point; the sensing model is the attenuated disk model.

**Figure 12:**
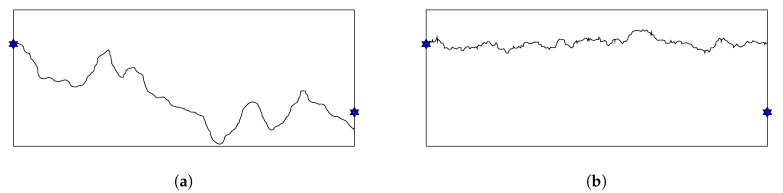
Penetration path created by (**a**) control-point initialization; (**b**) random initialization.

From Figure 11, we can see that the control-point initialization greatly surpasses the random initialization to obtain smaller minimal exposure value in case of Gauss distribution. On the other hand, there is no significant difference between two methods when sensors are uniformly distributed. Figure 12 illustrates a penetration path created by these two methods. While the path created by the control-point method looks rugged and can reach the far the top or the bottom of the ROI, the other one seems to flow horizontally from the left to the right. When sensors are distributed according to Gauss distribution, an intruder tends to travel along the top or bottom border since the density of sensor is high in the middle of sensing field. In this case, the control-point gives much better results than the random method. Whereas, when sensors are uniformly distributed in the ROI, there might be multiple paths that have low exposure and our HPSO-MMEP still runs effectively with the random initialization.

#### 4.2.2. Comparison between HPSO-MMEP and GAMEP [39]

We run our HPSO-MMEP algorithm and GAMEP [39] algorithm on various experimental instances to prove the efficiency of HPSO-MMEP over GAMEP. Parameters of GAMEP are set as in Table 5. Table 6 and Table 7 show the computation results of our HPSO-MMEP algorithm and the GAMEP algorithm in case of uniform distribution and Gauss distribution of sensors respectively. Table 6 and Table 7 indicate that:

- For the minimal exposure value, HPSO-MMEP wins over GAMEP in all experimental instance. The gap between the minimal exposure value achieved by the two algorithms is extremely large. For uniform distribution of sensors, the minimal exposure value obtained by HPSO-MMEP is on average 2.7 times smaller than one obtained by GAMEP. For Gauss distribution of sensors, the ratio is 2.1. The overall far better performance of HPSO-MMEP over GAMEP in various scenarios provides strong evidence of the effectiveness of our proposals for making the combination between GA and PSO, and making heuristics on the initialization of the population (control-point initialization). On the other hand, GAMEP is a pure genetic algorithm with the whole population generated from an initial individual. This causes lack of population diversity, which is one of the essential criteria for population-based meta-heuristics algorithm. As a result, GAMEP usually converges to a local optima, leaving a potential domain to get a better solution of the searching space uncovered.
- For standard deviation, HPSO-MMEP obtains a much smaller exposure than GAMEP in almost cases. Put Table 6 and Table 7 together: there are 32 experimental instances in total and we capture several statistical results. For our HPSO-MMEP algorithm, 25 out of 32 instances have standard deviations smaller than 2.0, while this fraction is only 8 out of 32 for the GAMEP algorithm. Among eight experimental instances in which GAMEP has a standard deviation smaller than 2.0, there are seven instances falls at N=25. This implies that GAMEP is stable only for a small number of sensors, and often fails to obtain stability when sensors are densely distributed. There are a few instances in which standard deviation of HPSO-MMEP is larger than GAMEP. However, except for the instance *g*_*t*_*rec*_100, there is only a slightly difference between standard deviation achieved by HPSO-MMEP and GAMEP at those instances; i.e., standard deviation in both algorithms is smaller than 1.0. In summary, HPSO-MMEP is highly stable since it obtains small standard deviations in almost all experimental instances and the standard deviation does not change significantly among those instances; i.e., standard deviation mostly falls on [0.0, 4.0]. Contrastingly, GAMEP has an extremely large standard deviation in some instances, and on average, the standard deviation of GAMEP is very high; i.e., 5.0 on average. The attractive results in standard deviation of HPSO-MMEP compared to GAMEP also suggest that HPSO-MMEP may approach close to global optima of the MMEP problem.
- For the computation time, HPSO-MMEP is longer than GAMEP in all instances. The correlation between the computation al time of the two algorithms is closed to linearity. This makes sense since both algorithms run in a fixed number of iterations. The extreme ratios between the computational time of HPSO-MMEP and GAMEP are 1.1 and 2.0, which is acceptable for the trade-off between the computation time and the solution accuracy and stability. Note that the complexity of the fitness calculation is the same in HPSO-MMEP and GAMEP. The difference in computation time comes from the speed of convergence of each algorithm. For HPSO-MMEP, it takes a longer time than GAMEP for the algorithm to converge. This again confirms that HPSO-MMEP explores the searching space of MMEP problem more thoroughly to give a better solution.

#### 4.2.3. Comparison between HPSO-MMEP and HPSO [33]

**Figure 13:**
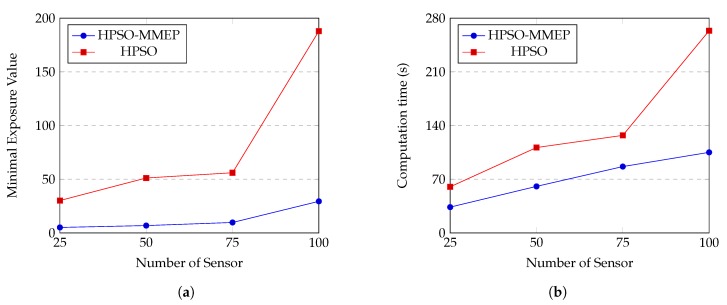
Comparison between HPSO-MMEP algorithm and HPSO algorithm regarding (**a**) the minimal exposure value; (**b**) the computation time when the number of sensor varies from 25 to 100.

Settings of HPSO are given in Table 8. We run our HPSO-MMEP algorithm and HPSO algorithm on the experimental instance *u*_*a*_*ran* and the results are shown in Figure 13. Note that HPSO deals with the MEP problem in a static sensor network. Hence, for the two algorithms to be comparable, we simply set value of $v_{S}$ in our MMEP model to zero. Experimental results show that our HPSO-MMEP algorithm outperforms HPSO in both the minimal exposure value and the computational time. Specifically, in Figure 13a, there is a remarkably big gap between HPSO-MMEP and HPSO with respect to the minimal exposure value. The higher value of *N* is, the bigger the gap in the minimal exposure value between the two algorithms is. Especially, the gap grows up a big distance between N=75 and N=100. Since HPSO-MMEP performs stably in almost cases, this might be due to the ineffectiveness of HPSO when dealing with complex experimental instances. For the computational time, Figure 13b shows that HPSO-MMEP obtains much better results than HPSO. We observe that the correlation between HPSO-MMEP and HPSO with respect to the minimal exposure value and computational time are quite the same. There is also a big jump in the gap between the computation time of HPSO-MMEP and HPSO from N=75 to N=100, which implies that HPSO is not stable in term of the computational time. Hence, when the number of sensors increases, HPSO poses disadvantages in both the solution accuracy and the computational time, which can be effectively addressed using our proposed HPSO-MMEP algorithm. Although both HPSO-MMEP and HPSO are inspired by the idea of combining genetic algorithm and particle swarm optimization, the key that makes HPSO-MMEP distinguished is the ability of vigorously searching, which is missing in HPSO. In this paper, HPSO-MMEP is proposed to solve the particular MMEP problem. However, HPSO-MMEP is not only restricted in the scope of this work but can also be applied to a variety of MEP models or even more general in the optimization problem with promise performance.

#### 4.2.4. Effects of Several Factors of Mmep Problem on the Solution

The minimal exposure value represents the lowest capacity to be detected of an intruder crossing the sensor network. This value also measures the network quality. This section studies the effects of several factors on the minimal exposure value: the speed $v_{I}$ of intruder and some network properties.

HPSO-MMEP is an approximate algorithm aiming at finding a solution as close as possible to the true solution. As analyzed so far, HPSO-MMEP is highly stable and is capable of effectively exploring the massive searching space of the MMEP problem. Hence, the minimal exposure value obtained from HPSO-MMEP can reliably reflect the network quality and we use this value to evaluate the network quality in all subsequent sections. Note that the network quality is independent from any algorithm; it depends only on the intrinsic properties of the network, such as the number of sensors, the topology of sensors and type of sensors.

*(a) Effect of intruder’s speed $v_{I}$*

**Figure 14:**
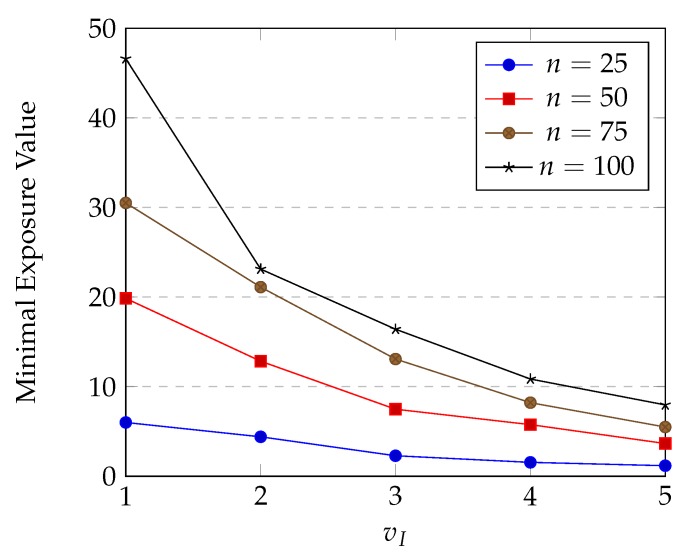
Effects of intruder’s speed on minimal exposure value.

Exposure of an intruder is expected to increase when time for which the intruder stays in the sensing field gets longer. Hence, it makes sense that exposure value is inversely proportional to the speed $v_{I}$ of the intruder. We run our HPSO-MMEP algorithm with different values of $v_{I}$ in several experimental instances to test the effect of this parameter on the minimal exposure value. The results are shown in Figure 14.

The experimental results completely matched our expectations. From this, we can conclude that our exposure definition is more appropriate compared to the traditional one.

Note that although the minimal exposure value varies when the speed $v_{I}$ of intruder varies, $v_{I}$ affects only to the capacity to be detected of the intruder, it does not reflect the network quality.

*(b) Effect of number of sensors N*

Experimental results in Table 6, Table 7 indicates that in all experimental scenarios, the network quality is improved when the number of sensors increases (recall that we used the minimal exposure value obtained from HPSO-MMEP to evaluate the network quality). For both Gauss distribution and uniform distribution of sensors, the minimal exposure value slightly rises between N=25 and N=50, but soars between N=75 and N=100. In some rare cases, the minimal exposure value is even lower when the number of sensors is larger, e.g., instances *g_t_rec_25* and *g_t_rec_50*, instances *u_t_rec_25* and *u_t_rec_50*. This implies that the number of sensors is not the only factor to affect to the network quality. The other factor is network topology. For the same the number of sensors, performance of the network might vary significantly when network topology changes. To summarize, we can conclude that the network quality depends largely on the number of sensors; however, the effect of the sensor number does not dominate other factors, typically topology of sensors in the ROI.

We also observe that there is no limitation for the minimal exposure value; i.e., the exposure value approaches infinity when the number of sensors approaches infinity. In practice, the minimal exposure value of a network higher than a threshold is sufficient to perform monitoring tasks. The selection of the threshold depends on the expectation of the network designer on the network performance.

*(c) Comparison between mobile sensor network and static sensor network*

**Figure 15:**
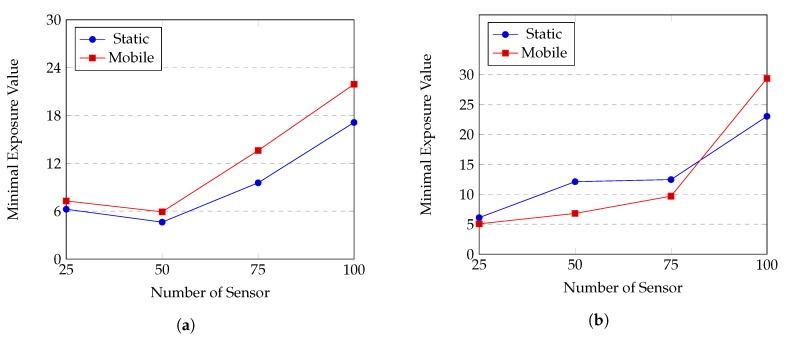
Performances of a mobile sensor network and a static sensor network: (**a**) Gauss distribution; (**b**) uniform distribution.

Network quality when using mobile sensors and static sensors are compared with each other on two sensor distributions, using the attenuated disk model and the random point sensor trajectory. The results are shown in Figure 15.

We see that for Gauss distribution, MWSN always performs better than SWSN, whereas, for uniform distribution, MWSN is often less efficient. The main reason behind is the effects of sensor movements. For Gauss distribution, initial positions of sensors form a network topology with many holes in coverage; i.e., the regions closed to the top or bottom border of the ROI are poorly covered. In this case, the movements of sensors help to fill up the coverage holes, thus improve the network quality. In contrast, for uniform distribution, coverage quality at every position in the ROI is relatively even, and sensor movements might cause new coverage holes and degrade the quality of sensor network. However, when the number of sensors is large (N=100), MWSN results in better performance for both Gauss and uniform distributions. This is because coverage holes no longer matter when *N* becomes large; i.e., there hardly exists coverage holes in the ROI both in static and mobile scenarios. In addition, expected coverage quality over time at almost positions in the ROI might be higher with the movements of sensors, thus, MWSNs obtain the expected performance of improving network coverage quality.

## 5. Conclusions

The MEP problem serves as an effective tool to evaluate the coverage quality of a sensor network. In this paper, we investigated the MEP problem in a mobile wireless sensor network (MMEP problem), which is a new and potential model that can offer a lot of additional advantages compared the traditional static MEP model. With the idea of combining the strengths of the genetic algorithm and particle swarm optimization, the HPSO-MMEP algorithm was devised to solve the MMEP problem. Different experimental instances were generated with the variation of the sensor regarding number, sensor distribution and sensor trajectories to examine our HPSO-MMEP algorithm. Effects of different factors on the performance of HPSO-MMEP algorithm and the network quality were thoroughly analyzed and explained after conducting numerous experiments. The results show that our algorithm successfully addresses the MMEP problem and is more efficient than previous algorithms. Furthermore, HPSO-MMEP is not restricted in the scope of this work as it is also applicable to a variety of MEP models or even general optimization problems.

## Figures and Tables

**Table 1:** Comparative table of related works on the MEP problem.

Publications	Method Used				Network Model	
	Voronoi Diagram	Grid-Based	Metaheuristic		Static	Mobile
[12,13,24,27,29,30]	√				√	
[25,26,28]	√	√			√	
[32]		√			√	
[31,35]		√	√		√	
[33,34,36,37]			√		√	
[38]	√					√
[39]			√			√

**Table 2:** Experimental parameters for attenuated disk model and truncated attenuated disk model.

Sensing Model	Parameter	Value
Attenuated disk model	Constant *C*	1
	Path attenuation exponent λ	2
Truncated attenuated disk model	Constant α	0.5
	Constant β	1
	Certain range $R_{1}$	1
	Sensing range $R_{2}$	10

**Table 3:** Experimental parameters for HPSO-MMEP algorithm.

Parameter	Value
Swarm size	100
Number of GA iterations	100
Number of PSO iterations per GA iteration	10
Acceleration coefficients	$C_{1}=0.8$
	$C_{2}=0.7$
	ω=1
Crossover rate	100%
Inverse mutation rate	30%
Symmetric mutation rate	70%

**Table 4:** Different versions of HPSO-MMEP using different genetic operators.

	HPSO-MMEP	HPSO-MMEP1	HPSO-MMEP2	HPSO-MMEP3	HPSO-MMEP4
Crossover	√	√	√		
Inverse mutation	√	√		√	
Symmetric mutation	√		√	√	

**Table 5:** Parameters setting for GAMEP [39].

Parameters	Value
Number of running on each instance	30
Population size	100
Number of iterations	200
Crossover rate	50%
Mutation rate	5%

**Table 6:** Computation results of HPSO-MMEP in comparison with GAMEP in uniform distribution of sensors (Mev: minimal exposure value, Sd: standard deviation).

Sensing Model	Trajectory	Num	HPSO-MMEP			GAMEP		
			Mev	Sd	Time (ms)	Mev	Sd	Time (ms)
Attenuated	Rectangle	25	4.0943	0.2752	33,976	7.3192	0.1889	20,970
		50	6.5169	0.6242	60,410	26.898	1.8234	37,516
		75	12.2150	0.23488	83,351	33.8265	3.8030	53,504
		100	26.2017	2.5010	92,361	45.0035	4.3400	75,283
	RanPoint	25	5.0750	0.3284	33,603	14.4034	2.0216	19,744
		50	6.8178	0.5703	60,269	21.1367	0.6702	42,236
		75	9.7004	0.18515	86,460	42.4746	2.3046	60,835
		100	29.3876	3.6232	105,081	41.7169	3.240	76,955
Truncated	Rectangle	25	3.2934	0.7606	34,828	8.3944	0.7621	18,973
		50	4.2928	0.1333	56,075	32.667	12.568	43,700
		75	14.5890	0.5100	78,390	43.26501	8.6802	61,154
		100	36.7583	1.8389	100,346	65.4624	6.6035	83,299
	RanPoint	25	6.4533	1.5017	36,802	19.2233	5.7220	25,598
		50	4.2184	0.3298	57,800	34.5452	3.1451	48,874
		75	4.4952	0.3681	83,477	48.8881	20.4467	68,922
		100	31.1390	3.7327	103,037	62.1939	4.6001	84,194

**Table 7:** Computation results of HPSO-MMEP in comparison with GAMEP in Gauss distribution of sensors (Mev: minimal exposure value, Sd: standard deviation).

Sensing Model	Trajectory	Num	HPSO-MMEP			GAMEP		
			Mev	Sd	Time (ms)	Mev	Sd	Time (ms)
Attenuated	Rectangle	25	3.7705	0.5323	38,815.7	6.3678	0.3921	20,252.9
		50	4.8387	1.5128	58,371.8	16.1140	3.07555	38,618.4
		75	14.6659	1.5564	76,501.1	21.6330	5.9376	55,233.2
		100	22.9836	2.0796	86,158	46.2440	2.63185	74,937
	RanPoint	25	2.5580	0.1083	38,155.9	8.5071	0.5490	20,359
		50	6.5290	0.4990	63,089	17.2894	3.7424	41,227.1
		75	10.7616	1.0230	85,041.9	30.2552	5.5275	61,047.1
		100	17.3493	3.9408	91,420.3	39.6380	6.6926	79,798.6
Truncated	Rectangle	25	2.1049	0.9336	37,528.7	8.0387	0.5067	18,565
		50	1.8612	2.5485	60,164.4	3.5942	3.7870	38,110.9
		75	20.5613	1.4335	77,512.4	27.3091	19.3064	57,500.7
		100	18.0101	9.7088	90,404.2	24.5270	3.9843	75,411.9
	RanPoint	25	0.6000	0.0691	37,441.5	11.9273	0.9837	20,707.8
		50	4.2179	0.5602	59,339.2	17.2173	3.0961	40,000.1
		75	7.4190	1.3398	81,291.4	26.2230	15.8393	61,232
		100	11.0246	1.2378	90,070.1	22.4935	3.1480	75,302.6

**Table 8:** Parameters settings for HPSO [33].

Parameters	Value
Number of running on each instance	20
Number of iterations	200
Swarm size	200
Acceleration coefficients	$C_{1}=C2=2$
	ω=1
Mutation rate	100%
